# Inhibition of PI3K/Akt/mTOR signaling in PI3KR2-overexpressing colon cancer stem cells reduces tumor growth due to apoptosis

**DOI:** 10.18632/oncotarget.9919

**Published:** 2016-06-08

**Authors:** Sugong Chen, Robert C. Fisher, Steven Signs, L. Alex Molina, Anitha K. Shenoy, Maria-Cecilia Lopez, Henry V. Baker, John M. Koomen, Yi Chen, Haley Gittleman, Jill Barnholtz-Sloan, Annamarie Berg, Henry D. Appelman, Emina H. Huang

**Affiliations:** ^1^ Department of Surgery, University of Florida, Gainesville, Florida 32610, USA; ^2^ Department of Stem Cell Biology and Regenerative Medicine, Cleveland Clinic Lerner Research Institute, Cleveland, Ohio 44195, USA; ^3^ Department of Molecular Genetics and Microbiology, University of Florida, Gainesville, Florida 32610, USA; ^4^ Molecular Oncology and Proteomics, SRB3, H. Lee Moffitt Cancer Center and Research Institute, Tampa, Florida 33612, USA; ^5^ Bioinformatics, Case Comprehensive Cancer Center, Cleveland, Ohio 44106, USA; ^6^ Department of Pathology, University of Michigan, Ann Arbor, Michigan 48109, USA

**Keywords:** colon cancer stem cells, ALDEFLUOR, tumorigenesis, PI3K/Akt/mTOR signaling, apoptosis

## Abstract

In sporadic colon cancer, colon cancer stem cells (CCSCs) initiate tumorigenesis and may contribute to late disease recurrences and metastases. We previously showed that aldehyde dehydrogenase (ALDH) activity (as indicated by the ALDEFLUOR^®^ assay) is an effective marker for highly enriching CCSCs for further evaluation. Here, we used comparative transcriptome and proteome approaches to identify signaling pathways overrepresented in the CCSC population. We found overexpression of several components of the phosphoinositide 3-kinase (PI3K)/Akt/mechanistic target of rapamycin (mTOR) signaling pathway, including PI3KR2, a regulatory subunit of PI3K. LY294002, a PI3K inhibitor, defined the contribution of the PI3K/Akt/mTOR signaling pathway in CCSCs. LY294002-treated CCSCs showed decreases in proliferation, sphere formation and self-renewal, in phosphorylation-dependent activation of Akt, and in expression of cyclin D1. Inhibition of PI3K *in vivo* reduced tumorigenicity, increased detection of cleaved caspase 3, an indicator of apoptosis, and elevated expression of the inflammatory chemokine, CXCL8. Collectively, these results indicate that PI3K/Akt/mTOR signaling controls CCSC proliferation and CCSC survival, and suggests that it would be useful to develop therapeutic agents that target this signaling pathway.

## INTRODUCTION

Colon cancer remains the third most common cancer in the US, and the third most common cause of cancer death [1]. While therapeutic approaches for locally advanced colon cancer have improved, treatments to prevent long-term disease recurrence and to mitigate metastatic disease are poor, ultimately resulting in a lower quality of life and decreased survival [2]. Currently available evidence suggests that 10-30% of patients have metastatic disease at the time of presentation.

One possible explanation for these oncologic challenges is the colon cancer stem cell (CCSC). These cells are a rare, self-renewing population within the epithelial tumor mass, constituting <10% of the total cells in the tumor [3]. As predicted by the cancer stem cell (CSC) hypothesis, incomplete elimination of the CCSC population permits continued growth of the cancer. To study CCSCs, several groups have enriched them from primary tumor tissues [4–6]. Indeed, we previously reported that the ALDEFLUOR^®^ assay enriches tumor cell suspensions for CCSC [6]. However, only limited progress has been made in developing treatment strategies that target CCSC [7].

In the current study, we used both transcriptomic and proteomic approaches to identify signaling pathways that can serve as targets for therapeutic interventions directed at disabling the CCSC population.

## RESULTS

### The PI3K/Akt/mTOR pathway in ALDEFLUOR^high^ CCSCs

We previously reported that differential fractionation of tumor xenografts based on ALDH enzymatic activity (ALDEFLUOR^®^ assay) represents an effective strategy to highly enrich collections of tumor cells for ALDEFLUOR^high^ CCSCs and ALDEFLUOR ^low^ progenitor cell subpopulations [6, 8]. As we demonstrate using *in vivo* limiting dilution analysis ALDEFLUOR^high^ CCSCs are functionally distinct from ALDEFLUOR ^low^ progenitor cells by virtue of their ability to self-renew, and maintain tumor initiating activity upon serial passaging in immunodeficient mice (Supplemental Figure S1). Compared to the stem cell properties of the ALDEFLUOR^high^ subpopulation, the ALDEFLUOR^low^ subpopulation behaved as progenitor cells, exhibiting limited self-renewal capacity affecting both the growth rate and number of secondary tumors (Supplemental Figure S1). This enrichment strategy was applied to a total of six different human sporadic colon cancers being maintained as tumor xenografts.

**Figure 1:**
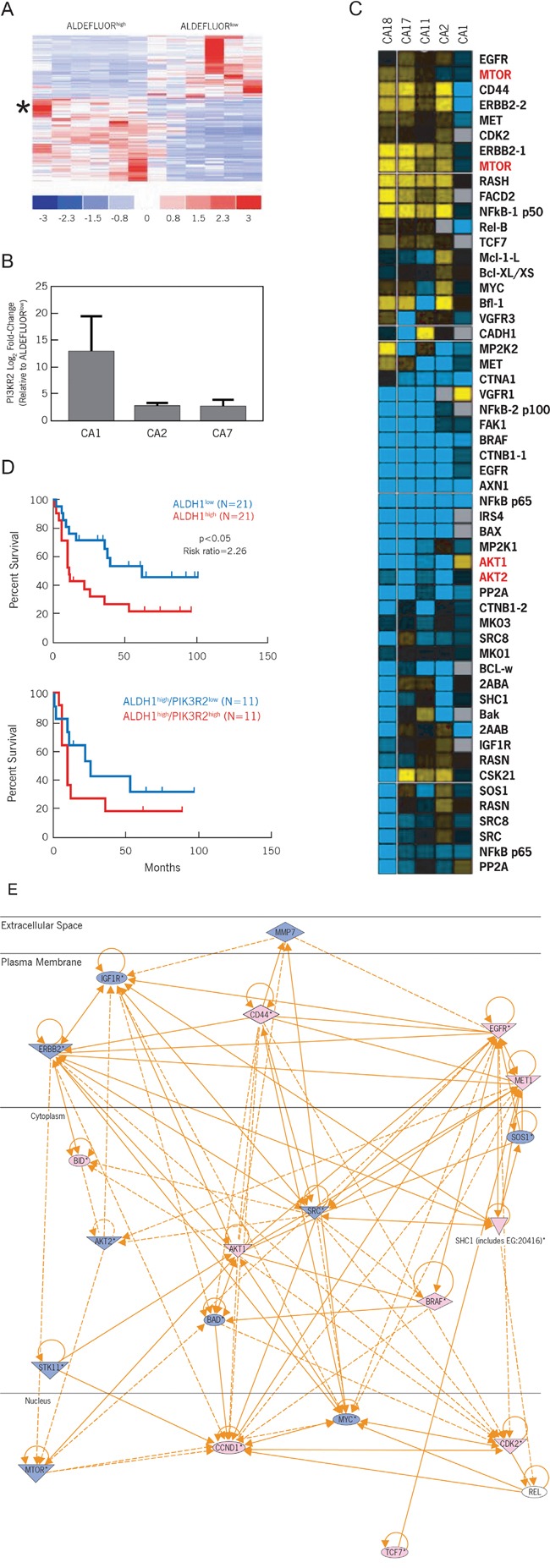
The PI3K/Akt/mTOR pathway is differentially expressed between ALDEFLUOR^high^ CCSCs and ALDEFLUOR^low^ progenitor cells **A**. Affymetrix gene array analysis of ALDEFLUOR^high^ versus ALDEFLUOR^low^ subpopulations. Sorted tumor xenograft cells from 6 different human colon cancer specimens revealed 136 genes that were significantly up- or down-regulated (p<0.001). PIK3R2 was upregulated 5.2-fold (star). **B**. Log_2_ fold expression differences between ALDEFLUOR^high^ vs. ALDEFLUOR^low^ samples sorted from tumor xenografts CA1, CA2 and CA7. **C**. Heatmap summary of liquid chromatography – multiple reaction monitoring mass spectrometry for selected signaling pathways and cellular processes. Log ratio values of ALDEFLUOR^high^ versus ALDEFLUOR^low^ subpopulations for each sorted tumor xenograft is demonstrated. Yellow: increased expression; Blue: decreased expression. **D**. Correlation of ALDH1 and PI3KR2 expression in patients with colon cancer. Kaplan Meier survival analyses were conducted using the Reid colorectal cancer patient database (Oncomine®). For the top panel (ALDH1 stratified as low and high), log rank p < 0.05, risk ratio is 2.26. For the bottom panel (ALDH1^high^ stratified by PI3KR2), log rank p = 0.3275, risk ratio is 1.64. **E**. Ingenuity pathways analysis. Superimposing the targeted proteomic analysis on the transcriptome analysis, the central node of activity suggests Akt and mTOR as potential effectors.

To further define and functionally understand the behavior of CCSCs, we queried potential signaling pathways using both transcriptomic and proteomic approaches to interrogate a panel of CCSCs being used to propagate tumor xenografts. The resulting matched ALDEFLUOR^high^ CCSC and ALDEFLUOR^low^ progenitor populations were used to generate cDNA-based targets for hybridizing to Affymetrix microarrays to define their gene expression profiles. Comparative gene expression analysis revealed 136 genes that were differentially expressed by ALDEFLUOR^high^ and ALDEFLUOR^low^ cells at a significance level of p<0.001 (Figure 1A, Supplemental Table 2). PI3KR2, one of the regulatory subunits that controls the catalytic subunit of PI3K, was upregulated 5.2-fold in ALDEFLUOR^high^ CCSCs. qRT-PCR analysis was used to confirm this finding for 3 of our sporadic colon cancers. The differences in PI3KR2 expression ranged from 3- to 13-fold (Figure 1B).

An orthogonal limited proteomics approach using liquid chromatography-multiple reaction monitoring mass spectrometry (LC-MRM) analysis was initiated to identify signaling pathways or associated processes (apoptosis and receptor-associated tyrosine kinase mediated phosphorylation) that regulate CCSCs [9]. These studies revealed greater differences in the expression of peptides that are associated with the PI3K/Akt/mTOR pathway (Figure 1C) including Akt and mTOR proteins. We therefore hypothesized that the PI3K/Akt/mTOR pathway controls CCSC functions.

To define the clinical significance of PI3K/Akt/mTOR signaling in ALDEFLUOR^high^ CCSCs, we initially queried the Oncomine^®^ database for the significance of ALDH1 overexpression in the Reid colorectal cancer patient data set (Compendia Bioscience, Ann Arbor, MI). In support of our hypothesis, colorectal cancer patients with increased ALDH1 expression exhibited increased mortality (Figure 1D, top panel; p < 0.05 and a risk ratio of 2.26). Further stratification of ALDH1^high^-expressing colon carcinomas based on PI3KR2 expression indicated a shorter lifespan in patients overexpressing PI3KR2 (Figure 1D, bottom panel; risk ratio of 1.64). Therefore, increased PI3KR2 expression can potentiate further activation of PI3K and downstream mediators (Ingenuity Systems Pathway Analysis; version 6; Figure 1E).

### Inhibition of PI3K results in decreased proliferation and decreased self-renewal *in vitro*

**Figure 2:**
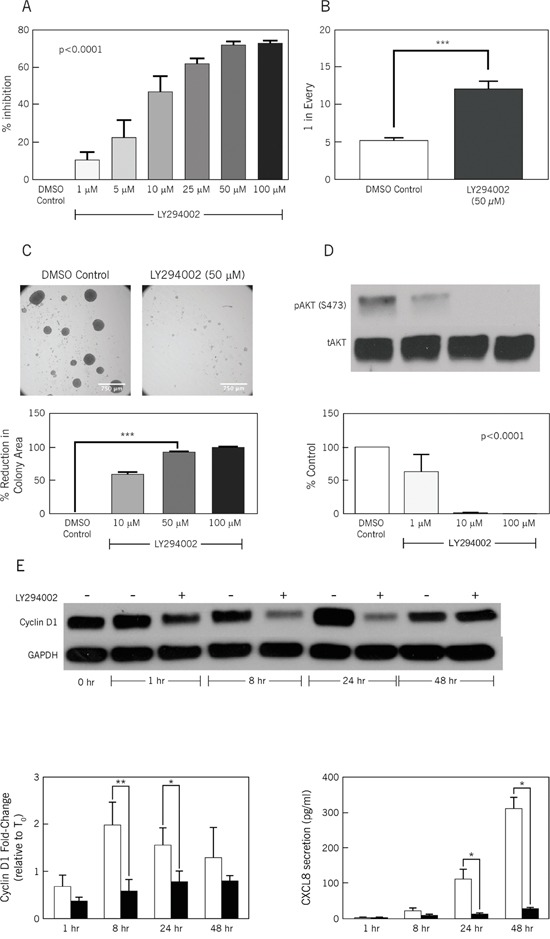
LY294002, a PI3K inhibitor, inhibits stem cell frequency and proliferation **A**. BrdU Proliferation Assay in the presence of increasing doses of LY294002 (0-100 μM). Y-axis: relative light units per second, a measure of BrdU incorporation, represented as percentage inhibition compared to 0 μM (DMSO) control. X-axis: increasing drug concentration. (n=6, p<0.0001, one-way ANOVA). **B**. Limiting Dilution Assay: ALDEFLUOR^high^ CCSCs (CA2) with and without LY294002 treatment. Y-axis: frequency of sphere formation. One in approximately 5 control cells formed spheres versus one in 12-14 treated cells. (n=6, _**_*p<0.0001, Student's t-test). **C**. Colony Formation Assay: Upper panels demonstrate relative size and number of colonies in the presence of the control (DMSO) vs. the PI3K inhibitor, LY294002. The lower panel demonstrates percent reduction in total colony area with increasing doses of LY294002, compared with DMSO control; Size bar: 750 μm. (n=6, _**_*p<0.0001, one-way ANOVA). **D**. Western blotting for pAkt: Increasing concentrations of LY294002 treatment (20 minute treatment) resulted in augmented inhibition of Akt phosphorylation at serine 473. The graph shows densitometry of pAkt (serine 473) normalized against total AKT (n=3, p<0.0001, one-way ANOVA). **E**. Western blot for total Cyclin D1 expression by LY294002 (50 μM)-treated CA2 CCSCs at 1, 8, 24 and 48 hours. Left graph shows densitometry of Cyclin D1 normalized against GAPDH (n=3 to 4, *p<0.05, **p<0.01, Student's t test). Right graph show CXCL8 secretion levels (pg/mL) at 1, 8, 24 and 48 hours for LY294002 (50 μM) treated-CA2 CCSCs (n=3 to 4, * = p<0.05, Student's t test).

We chose the CA2 CCSC sphere isolate to investigate the *in vitro* effects of LY294002, a PI3K inhibitor, and rapamycin, an mTOR inhibitor. Incorporation of BrdU revealed a dose-dependent inhibition by LY294002 of CCSC proliferation (Figure 2A; p<0.0001). Maximum inhibition by LY294002 was detected at 50 μM, which was chosen for subsequent *in vitro* studies. In contrast, rapamycin exhibited limited inhibition of BrdU incorporation (Supplemental Figure S2). Based on these initial findings, we focused on the PI3K inhibitor, LY294002. *In vitro* limiting dilution assays were used to measure the effect of LY294002 on CCSC self-renewal. LY294002 treatment significantly decreased self-renewal as indicated by a 2.4 fold decrease in frequency of sphere formation (Figure 2B; p<0.0001). As a second measure of proliferation, colony formation was assessed. LY294002-treated CA2 CCSCs had a 91.8% decrease in colony area (Figure 2C; 50 μM LY294002; p<0.0001).

### Phosphorylation of Akt is characteristic of PI3K activation

Western blotting was used to monitor phosphorylation of serine 473, which is known to activate Akt functions [10]. Treating CA2 CCSCs for 20 minutes with increasing concentrations of LY294002 significantly decreased serine 473 phosphorylation (Figure 2D; p<0.0001; Supplemental Figure 3A and B), and therefore validated the potential of LY294002 to inhibit Akt activity.

### Activated PI3K regulates cyclin D1 and CXCL8

We tested the functional importance of PI3K signaling in regulating cyclin D1 and CXCL8 levels in CCSCs by treating CA2 cells with 50 μM Ly294002 for 1, 8, 24 or 48 hours. These times were chosen to document the oscillating levels of cyclin D1 as the CA2 CCSCs progress through G1 [11]. Cyclin D1 levels were measured by western blotting and CXCL8 levels were assayed by ELISA. Decreases in cyclin D1 protein levels were noted at 8 and 24 hours post-treatment (Figure 2E; bottom left; p<0.01 for 8 hours, p<0.05 for 24 hours; Supplemental Figure 3C and D). By 48 hours, there was no significant difference in cyclin D1 protein levels. For secreted CXCL8, significant decreases were observed at 24 and 48 hours post-treatment (Figure 2E; p<0.05 at 24 and 48 hours).

### Effect of PI3K inhibition on CCSC proliferation *in Vivo*

**Figure 3:**
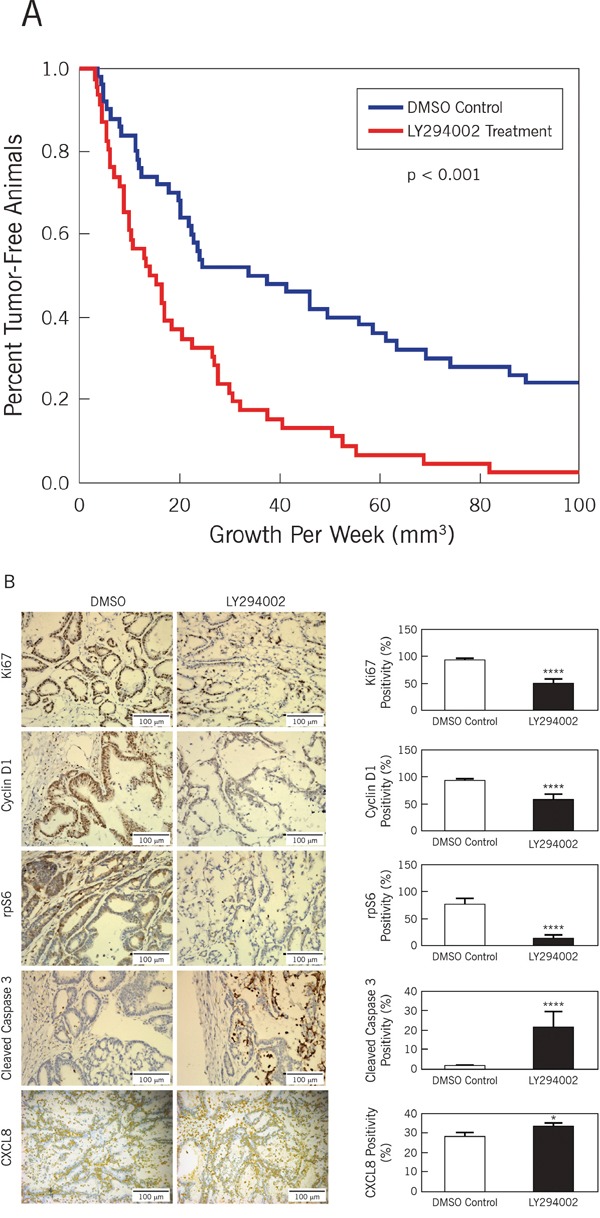
Inhibition of PI3K diminishes growth of CCSCs *in vivo* Tumor growth curve and immunohistochemistry of resulting xenografts in the presence and absence of LY294002. **A**. Tumors were initiated with 1000 CCSCs and allowed to grow until the volume was at least 3 mm^3^. Comparisons were made between groups receiving either drug carrier alone (DMSO) or LY294002 treatment. In the presence of LY294002, tumors were smaller and had increased tumor latency. Kaplan-Meier curves were generated to plot tumor growth (log-rank test, p < 0.001) **B**. Immunochemistry on xenograft control (DMSO) vs. LY294002 for Ki67, cyclin D1, phosphorylated rpS6, activated caspase3 and CXCL8. Representative images are displayed on the left and graphical enumeration of stained cells/total cells are graphed on the right. Ki67 is associated with cellular proliferation, cyclin D1 is associated with active cell cycling, phosphorylated rpS6 is a downstream effector initiating protein translation during PI3K activation, and cleaved caspase 3 is associated with apoptosis. Size bar = 100 microns.* p<0.05, **** p<0.0001.

Given the *in vitro* findings, we proceeded to examine the effect of LY294002 on CCSCs *in vivo*. Consistent with the clinical context, we evaluated the potential for PI3K inhibition to reverse tumorigenicity initiated by the CCSCs. Subcutaneous ALDEFLUOR^high^ tumor xenografts were established. Once the tumor was palpable at approximately 3 mm, the animals were divided into two groups receiving either DMSO alone or LY294002 at 50 mg/kg in DMSO. LY294002 treatment resulted in increased tumor latency and significantly reduced tumor growth (Figure 3A, p < 0.001).

Key components of the PI3K/Akt/mTOR signaling pathway were then evaluated by immunochemistry (Figure 3B). As expected, the expression of Ki67 was reduced by LY294002 treatment (p < 0.0001). Likewise, cyclin D1 showed decreased expression. Phosphorylation of rpS6, a key regulatory event downstream of mTOR activation and involved in the regulation of protein synthesis, was significantly reduced (p < 0.0001). Conversely, immunohistochemical staining for the apoptosis marker, cleaved caspase 3, was increased by LY294002 treatment (p < 0.0001).

### CXCL8 expression is increased in the tumor xenografts treated with the PI3K inhibitor, LY294002

We previously showed that anti-CXCL8 antibody treatment inhibited CCSC-induced intestinal tumorigenicity [8]. We therefore queried whether expression of CXCL8 in LY294002-treated tumors was affected. We found increased CXCL8 expression in the tumors treated with LY294002 (p < 0.05; Figure 3B, lower panels). In contrast to these *in vivo* findings, secreted CXCL8 levels in short-term CCSCs cultures treated with 50 μM Ly294002 were profoundly reduced throughout the 48 hour time course (Figure 2E).

## DISCUSSION

CSCs have been reported in multiple solid organ malignancies including colon cancer and have been associated with both metachronous recurrences and metastases [12]. Therefore, to maximize the efficacy of therapeutics, CSCs must be targeted.

To better define therapeutic targets for CCSCs, a two pronged strategy was undertaken to identify signaling pathways selectively overexpressed in ALDEFLUOR^high^ CCSCs versus ALDEFLUOR^low^ progenitor cell populations. Data sets generated from transcriptome analysis and a complementary proteomic approach were compared which identified several components of the PI3K/Akt/mTOR signaling pathways as being highly expressed in the ALDEFLUOR^high^ CCSC subpopulation (Figure 1E). Prompted by the observation that PI3KR2 is overexpressed in breast and colon carcinomas, and correlates with PI3K activation and tumor progression [13], we confirmed that PI3KR2 is significantly overexpressed in our panel of ALDEFLUOR^high^ CCSC samples (Figure 1B), Furthermore, colorectal cancer patients expressing high levels of both the CCSC marker, ALDH1, and PI3KR2 show a reduced survival (Figure 1D). Collectively, these findings demonstrate the potential for the PI3K/Akt/mTOR pathway to control ALDEFLUOR^high^ CCSC functions.

Our initial *in vitro* functional studies using the PI3K and mTOR inhibitors, LY294002 and rapamycin, respectively, identified a functional hierarchy with LY294002 having a greater ability than rapamycin to inhibit CCSC proliferation (Figure 2A and Supplemental Figure 3). These initial results reflect the pivotal role of PI3K in controlling the phosphorylation of intracellular inositol lipids, which in turn activates effector molecules such as Akt [14]. Activation status of Akt represents an important checkpoint affecting cell metabolism, cell cycle regulation, and apoptosis, all of which have been implicated in cancer initiation and progression. The functional importance of PI3K is further substantiated by the correlation of PI3K mutations with the development of cancer [15, 16]. We observed a consistent linkage between PI3K inhibition and AKT activation (Figure 2D), cyclin D1 levels (Figure 2E), CCSC proliferation (Figure 2A and 2C) and self-renewal (Figure 2B) *in vitro*. Likewise, in tumor reversal experiments, PI3K inhibition resulted in suppressed tumorigenesis. The inhibition of proliferation appears to be due to apoptosis as evidenced by increased levels of cleaved caspase 3 in the LY294002-treated xenografts (Figure 3A and 3B). Other studies using CD133 [17] or ALDH1B1 [18] expression to enrich for CCSCs, corroborate our findings that the PI3K/Akt/mTOR pathway contributes to the pathogenesis of colon tumorigenesis.

Though there is a clear link between inflammation and cancer, the mechanism of how inflammation promotes cancer is still poorly understood [19]. We and others have previously demonstrated that CXCL8 is upregulated and functionally important in colorectal tumorigenesis due to its ability to promote cell proliferation, cell survival and angiogenesis [8, 20]. A link between CXCL8 and the cell cycle has been reported, which further clarifies the mechanistic role of CXCL8 in colon cancer [21, 22]. These studies have demonstrated that CXCL8 signaling is required for G1 to S progression for both prostate and breast cancer.

Our *in vivo* tumor reversal experiments using the PI3K inhibitor, LY294002, demonstrated not only tumor regression via apoptosis but also a state of chronic inflammation as evidenced by the increased expression of the inflammatory chemokine, CXCL8. The expression of CXCL8 may be the result of tumors undergoing senescence and expressing a senescence–associated secretory phenotype (SASP), characterized in part by the secretion of inflammatory factors including CXCL8 [23]. Alternatively, cellular debris from dying tumor cells may function as damage-associated molecular pattern (DAMP) molecules. The resulting DAMP molecules are capable of binding and triggering toll-like receptors, thereby inducing the expression of interleukin 1β and tumor necrosis factor-α with subsequent expression of NF-κB target genes, including CXCL8 [24]. For either scenario, there exists the possibility of CXCL8 promoting the growth of the tumor. In contrast, our *in vitro* LY294002 inhibition experiments do not demonstrate increased secretion of CXCL8. One plausible explanation is that the short duration of the studies is insufficient to display SASP or induce a DAMP-mediated response.

**Figure 4:**
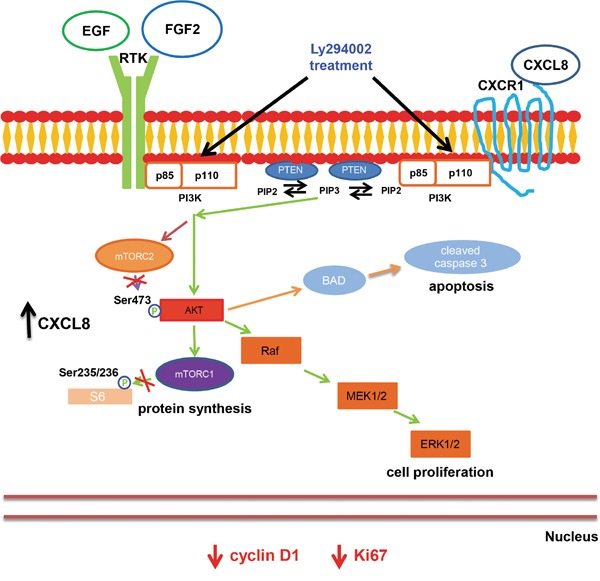
Model CCSCs possess an activated PI3K-Akt-mTOR pathway. Continuous treatment with Ly294002, a PI3K inhibitor, diminished proliferative indices and cyclin D1 expression, and increased cleaved caspase 3, indicative of an active apoptosis cascade resulting in an overall decrease in tumorigenicity. Increases in CXCL8 expression may promote survival and proliferation of a CCSC population that is refractory to LY294002 treatment.

Our research used a well-studied inhibitor of PI3K activity, LY294002, to confirm that the PI3K/Akt/mTOR signaling pathways represents a viable therapeutic target for inhibiting CCSCs (Figure 4). However, the high degree of emerging resistance to single agent-based therapies warrants the use of multimodality therapeutic strategies. While beyond the scope of the current studies, potential multimodal treatments aimed at the CCSCs could be directed at both a key signaling pathway, such as the PI3K/Akt/mTOR pathway, and a complementary upstream activator such as the chemokine, CXCL8.

## MATERIALS AND METHODS

### Human specimens and cell line authentication

Tissues from patients with sporadic colon cancer were retrieved under pathologic supervision with Institutional Review Board approvals at the University of Florida and the University of Michigan. To insure the unique identity of the isolates, short tandem repeat analysis (DDC Medical, Fairfield, Ohio; Duke University DNA Analysis Facility, Durham, North Carolina) was completed at the time of successful engraftment, and, for *in vitro* CCSC sphere cultures, on initiation and at every fifth passage.

### Animals

All animal experiments in the study were performed with non-obese diabetic, severe combined immunodeficiency, IL2γ receptor null mice (NSG^®^ mice, Jackson Laboratory; #005557; males; 28 to 30 grams), following protocols approved by the University of Florida and the Cleveland Clinic Lerner Research Institute Institutional Animal Care and Use Committees. All mice were maintained under pathogen-free conditions with food and water ad libitum. Patient-derived tumor engrafts were generated as previously described [6]. *In vivo* limiting dilutions assays were used to confirm the self-renewal potential of CCSC populations enriched for high levels of ALDEFLUOR^®^ positive cells (Supplemental Figure S1).

### Flow-assisted cytometric sorting

Human epithelial cells were enriched from previously established human colon cancer xenografts by flow-assisted cytometric sorting (FACS) using antibodies binding human epithelial-specific antigen (ESA; Miltenyi Biotec; #130-091-254) and murine H-2K^d^/H-2D^d^ (Southern Biotech; #1911-08). The resulting H-2K^d-^/H-2D^d-^/ESA^+^ cells were injected subcutaneously into the flanks of mice. The resulting tumors were again dissociated and resorted as above using H-2K^d^/H-2D^d^- and ESA-specific antibodies plus the ALDEFLUOR^®^ kit (Stemcell Technologies; # 01700) for further fractionation into ALDEFLUOR^high^ and ALDEFLUOR^low^ cell subpopulations. All sorting was done using an Aria II instrument (BD Biosciences) with FACSDiva, Version 6.1.2 software (BD Biosciences)

### Expression profile analysis

Six different sporadic human colon cancers were used (Supplemental Table 1) to generate tumor xenografts for dissociation and sorting into ALDEFLUOR^high^ and ALDEFLUOR^low^ subpopulations as described above. Total RNA was isolated from sorted cells using Qiagen RNeasy Microkit (# 74034) and quality validated by capillary electrophoresis (Aligent 2100 Bioanalyzer). RNA samples with an RNA integrity value >7.0 were processed into double-stranded cDNA, and amplified using a WT-Ovation™ Pico RNA Amplification System V1 kit (NuGEN Technologies; #3300-12). Amplified cDNA samples were fragmented and biotin labeled using a FL-Ovation™ cDNA Biotin Module V2 kit (NuGEN Technologies; #4200-12). Labeled cDNA fragments were hybridized onto a Human Genome U133 Plus 2.0 GeneChip™ Probe Array (Affymetrix; #900466) using a fluidic station followed by washing and staining according to the manufacturer's recommendations. Stained arrays were scanned and the resulting images corrected for background and normalized in dCHIP (http://www.hsph.harvard.edu/cli/complab/dchip/) to generate a collection of probe sets corresponding to 54,675 genes. BRB-ARRAY Tools (version 4.3.0 Table Release; http://linus.nci.nih.gov/BRB-ArrayTools.html) was used to identify genes differentially expressed between ALDEFLUOR^high^ and ALDEFLUOR^low^ groups at a significance level of <0.001, and further confirmed by leave-one-out-cross-validation studies and Monte Carlo simulations (BRB-Array Tools).

### Proteomics

Pellets from the ALDEFLUOR^high^ and ALDEFLUOR^low^ cell populations were fractionated using cell sorting from patient-derived tumor xenografts as above. Equal amounts of protein from each population were further fractionated by SDS-PAGE, followed by disulfide reduction, cysteine alkylation, and in-gel digestion with trypsin. Peptides were extracted and concentrated with vacuum centrifugation; stable isotope-labeled standards and single conservative amino acid replacement peptides were spiked into each sample to enable quantification of endogenous peptide levels [25]. Liquid chromatography-multiple reaction monitoring mass spectrometry (LC-MRM) as previously reported [9] was used to quantify components present in a number of functionally important signaling pathways (BCR-Abl, Wnt, Notch, TGF-β/SMAD/BMP, Ras/Raf/MEK/ERK, PI3K/Akt/mTOR and NF-κB) or cellular processes (Receptor Tyrosine Kinase substrates and apoptosis) that are operative in model cancer cell lines [9, 26, 27]. Peptide separations were performed with trapping and analytical columns (Acclaim® PepMap100 C18; Dionex) using a nanoflow liquid chromatography system (UltiMate 3000; Dionex) operating at a flow rate of 250-300 nL/min with 45 minute gradient programs. Instrument settings for data acquisition on the triple quadrupole liquid chromatography mass spectrometer (TSQ Vantage®, Thermo Scientific) include spray voltage 2,500 V, Q1 resolution 0.4, Q3 resolution 0.7, and 1.5 mTorr Argon collision gas. Data were quantified using the sum of all transitions, normalized for GAPDH expression, and relative concentrations calculated using the ratio of endogenous tryptic peptide to the corresponding standard. Protein expression comparisons between ALDEFLUOR^high^ and ALDEFLUOR^low^ cells were expressed as log-ratio values, and visualized using Cluster 3.0 and Java TreeView.

### Cell culture

The human CCSC, denoted as CA2 and used in this study based on ability to be reproducibly propagated *in vivo* and *in vitro* as a sphere isolate (Supplemental Table 1). Briefly, this CCSC isolate was serially passaged as a tumor xenograft for at least 5 passages and enriched for ALDEFLUOR^high^ cells as described above. The resulting ALDEFLUOR^high^ cells were propagated in serum–free media (defined media; DM) containing fibroblast growth factor 2 (FGF2; 20 ng/ml) and epidermal growth factor (EGF; 40 ng/mL) using low attachment multi-well tissue culture plates [6, 8].

### CXCL8 quantification using ELISA

Culture medium was centrifuged at 1,200 rpm for 5 minutes at 37°C, and the supernatant collected and CXCL8 determined using a CXCL8 ELISA kit (RayBiotech; #ELH-IL8) according to the manufacturer's instructions.

### Limiting dilution sphere formation assay

Trypsin-dissociated CCSCs were plated by flow cytometry at increasing cell densities into DM containing either DMSO or LY294002 (50 μm; Calbiochem; #440202) as previously described [28]. Spheres were enumerated on day 7, and frequency was determined by Extreme Limiting Dilution Analysis “limdil” function (http://bioinf.wehi.edu.au).

### BrdU proliferation assay

Trypsin-dissociated CCSCs were plated in triplicate at 2,500 cells/100 μL DM into 96-well ViewPlate microplates (Perkin Elmer; cat. # 6005182) containing EGF, FGF2 and either DMSO, LY294002 (1 μM, 5 μM, 25 μM, 50 μM, 100 μM) or Rapamycin (20 nM, 100 nM, 500 nM). Following 5 days at 37°C and 5% CO_2_, cell proliferation was measured using a BrdU ELISA (Roche Diagnostics; # 11 669 915001) according to the manufacturer's instructions. Results were expressed as % inhibition relative to the DMSO control

### Methylcellulose colony formation assay

Single cell suspensions of trypsinized CCSCs were mixed with 2% methylcellulose (prepared in DMEM/F12; Invitrogen; # 12400-024) and DM supplemented with FGF2 (20 ng/mL), EGF (40 ng/mL) and DMSO or LY294002 (10 μM, 50 μM and 100 μM) for a final concentration of 1.0% methylcellulose. We then plated 5000 cells in a volume of 1.25 mL in triplicate into 3.5 cm non-tissue culture petri dishes, and subsequently incubated the cells at 37°C and 5% CO_2_ for 14 days. Five random images were documented per plate using a Leica DM1600 inverted microscope at 5X magnification. Total colony area was measured using ImageJ software and expressed as % reduction in colony area relative to the DMSO control.

### Western blotting

Cells from DMSO or LY294002 (1 μM, 10 μM, 50μM or 100 μM) treated CCSC cultures were harvested, washed with 1x D-PBS and lysed in RIPA lysis buffer (Sigma-Aldrich, # R0278) containing 1X protease inhibitors (Roche Diagnostics; # 0589791001) and 1X PhosSTOP (Roche Diagnostics; # 04 906 845 001). Proteins in resulting cell lysates were separated by SDS-PAGE (7.5%; #456-1024 or 4-15%; #456-1083 Mini-PROTEAN TGX Gels; Bio-rad Laboratories) and electrotransferred to a PVDF membrane (Immobilon-P membrane, Millipore; #IPVH00010). Following blocking with 5% nonfat dry milk (NFDM) in 1X TBS containing 0.1% Tween-20, membrane was incubated overnight at 4°C with either an anti-Phospho-Akt (Serine 473) rabbit mAb (Cell Signaling Technology; # 193H12; 1:1000) or an anti-human Cyclin D1 rabbit mAb (abcam; # ab16663; 1:250) in TBS-T with 1% BSA. Following washing with 1X-D-PBS, specifically bound antibody was detected by incubating washed membrane with goat anti-rabbit IgG-HRP secondary antibody (Sigma; #A0545; 1:40,000) for 1 hour at 25°C in TBS-T containing 5% NFDM, washing multiple times with TBS-T, and subsequently visualizing with an enhanced chemiluminescence detection system (ECL2 Western Blotting Substrate; Pierce, GE Healthcare; # 80196). To detect total protein or total Akt proteins, blots were treated with stripping buffer (# 21059; Thermo Fisher Scientific) according to the manufacturer's specifications, reblocked with TBS-T containing 5% NFDM and reprobed with an anti-GAPDH rabbit mAb (Cell Signaling Technology; # 2118; 1:40,000) or an anti-pan Akt rabbit mAb (Cell Signaling Technology; #C67E7; 1:1000) respectively, in TBS-T containing 1% BSA, and processed further as described above for other rabbit mAbs. Protein levels were quantified from scanned chemilumenescent images using ImageJ software. Levels of phospho-Akt were normalized to total Akt and cyclin D1 levels were normalized to GAPDH.

### *In vivo* inhibition of PI3K in CCSC-initiated tumor xenografts

Tumors were generated via flank subcutaneous injections of CCSC cell suspensions into mouse flanks. The suspensions were mixed with Matrigel^®^ (100 μL/injection; 1:1 mixture; BD Biosciences, #356234). Resulting tumors were harvested, dissociated and sorted for H-2K^d-^/H-2D^d-^, ESA^+^ and ALDEFLUOR^high^ as described above. Fifteen NSG mice received bilateral injections of 10^3^ H2K^d-^/H-2D^d-^ESA^+^ ALDEFLUOR^high^ CCSCs. Once the tumors reached 3 mm^3^, animals were divided into two groups that received either the drug vehicle (DMSO) alone or DMSO containing 50 mg/kg of LY294002 (LC Laboratories; #L-7962). Tumor size was measured twice weekly. All animals were sacrificed when their tumors reached 6-8 mm along the longest diameter. The tumors were excised and fixed in paraformaldehyde for subsequent immunochemical analysis.

### Immunohistochemistry

Paraformaldehyde-fixed tumor sections were deparaffinized in xylene and rehydrated in descending percentages of ethanol, and processed through an antigen retrieval step using Target Retrieval Solution (Dako; #S1699). After washing steps, slides were incubated with 3.0% hydrogen peroxide for 20 minutes at room temperature and rinsed thoroughly with water. Blocking was accomplished with 3% horse serum (Vector Laboratories; #S-2000) for 20 minutes at room temperature followed by rinsing briefly and incubating with Avidin D blocking solution for 15 minutes at room temperature (Vector Laboratories; #SP-2001) After a brief washing step, sections were incubated overnight with a primary antibody at 4°C. The following primary antibodies were used: Ki67 (DAKO; # M7240; 1:250), cleaved caspase-3 (Cell Signaling Technology; # 9664; 1:800), Cyclin D1 (Cell Signaling Technology; # 2978; 1:200), phosphorylated rpS6 (Cell Signaling #4858, 1:500) and CXCL8 (R&D Systems; cat. # MAB208; 1:150). Sections were washed, incubated with a secondary biotinylated anti-mouse IgG (Vector Laboratories; #BA-2001; 1:200) for 30 minutes at room temperature, washed, treated with a peroxidase reagent (VECTASTAIN ABC Elite kit; Vector Laboratories; #PK-6100) for 30 minutes at RT, washed, treated with Vector ImmPACT DAB Peroxidase Substrate (Vector Laboratories; #SK-4105) for up to 10 min, rinsed in water, counterstained in Vector Hematoxylin (Vector Laboratories; #H-3401) and rinsed a final time in water. After dehydration; the slides were coverslipped with Permount^®^ mounting medium. Representative images were documented at 20X using a Leica DM 4000B upright microscope or a Leica SCN400 F slide scanner. For each antibody, six DMSO-treated (control) tumors and six LY294002-treated tumors were quantified in which 2000 epithelial cells were counted per group. Positivity was quantified as the ratio of DAB-stained colonic epithelia to the total number of nuclei-positive colonic epithelia.

### Quantitative real-time polymerase chain reaction

To quantify the expression level of PI3KR2 in patient-derived tumor xenografts, human ESA^+^, murine H-2K^d-^/H-2D^d-^ cells were enriched further for ALDEFLUOR^high^ versus ALDEFLUOR^low^ cell subpopulations, and total RNA was isolated using RNeasy Plus minikits (Qiagen; #74134). The integrity of the RNA was confirmed by bioanalyzer analysis (Agilent 2100 Bioanalyzer). Total RNA underwent RNase–free DNase I treatment using either on-column treatment (Qiagen; #79254) or Amplification grade DNase I (Invitrogen Life Science Technologies, #18068-015) and processed into cDNA using iScript^®^ cDNA Synthesis Kit according to the manufacturer's specifications (Bio-Rad; #1708891). The resulting amplified cDNA underwent an additional round of amplification using the Ovation Pico WTA System V2 (NuGEN; #3302-60-NUG). TaqMan expression assays from Applied Biosystems were used to detect PI3KR2 (.Hs00178181_m1, phosphoinositide-3-kinase, regulatory subunit 2 beta) and the house keeping gene, IP08 (Hs00183533_m1), and analyzed on an Applied Biosystems Model 7500 or 7900 Real Time PCR instrument using SDS Version 2.3 and 2.4 software, respectively. Ct values were normalized using the IPO8 housekeeping gene and underwent comparative CT analysis using the 2^ΔΔCT^ method [29]. PI3KR2 expression by the ALDEFLUOR^high^ cell population was displayed as log_2_ fold-change relative to the ALDEFLUOR^low^ cell population.

### Statistics

Data are shown as mean ± SD. Paired Student's t-test or one-way ANOVA were used for comparisons of gene or protein expression measurements. Statistical significance was defined as p < 0.05 in all experiments except for gene expression analysis using microarray, for which, significance was defined as p < 0.001, in order to help control for multiple comparisons. Oncomine^®^ data sets were analyzed by Cox Proportional Hazards Survival regression analysis (http://statpages.org/prophaz.html). For tumorigenicity studies, Kaplan-Meier analysis was used to plot tumor growth per week using a threshold cutoff of 3 mm^3^. The difference between control and LY294002 treatment groups was determined using the log-rank test.

### Database accession number

The data discussed in this publication have been deposited in NCBI Gene Expression Omnibus and accessible through GEO Series accession number GSE70915.

### Figures

Digital images were cropped in Adobe Photoshop CS6. Final figures were constructed using Adobe Illustrator CS6.

## SUPPLEMENTARY MATERIALS FIGURES AND TABLES





## Abbreviations

ALDH: aldehyde dehydrogenase
CSC: cancer stem cell
CCSCs: colon cancer stem cells
DAMP: damage-associated molecular patterns
DM: defined media
EGF: epithelial growth factor
FGF2: fibroblast growth factor 2
IL1-β: interleukin 1-β
LC-MRM: liquid chromatography-multiple reaction monitoring mass spectrometry
mTOR: mechanistic target of rapamycin
NFDM: nonfat dry milk
PI3K: phosphoinositide 3-kinase
rpS6: ribosomal protein S6
SASP: senescence-associated phenotype.
