# Supplementary material for: Inhibition of PI3K/Akt/mTOR signaling in PI3KR2-overexpressing colon cancer stem cells reduces tumor growth due to apoptosis
Source: Oncotarget. 2016 Jun 8;8(31):50476–88. doi: 10.18632/oncotarget.9919 (PMC5584153; doi:10.18632/oncotarget.9919)
Supplement: Supplementary file 1 [file oncotarget-08-50476-s001.pdf]

Inhibition of PI3K/Akt/mTOR signaling in PI3KR2-overexpressing colon cancer stem cells reduces tumor growth due to apoptosis

SUPPLEMENTARY FIGURES AND TABLES

A

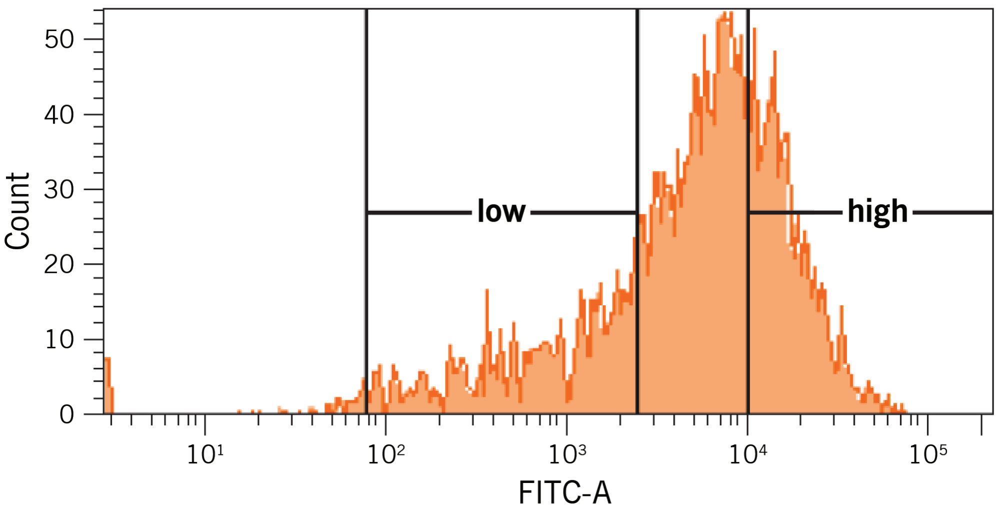

B

CA2 2°	ALDEFLUOR^{high}	ALDEFLUOR^{low}
100 cells	10/10	6/10

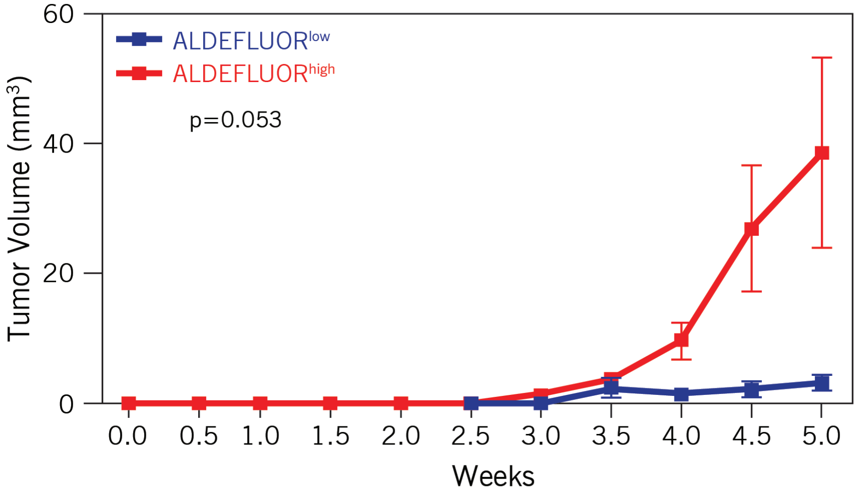

Supplementary Figure S1: ALDEFLUOR expression and *In vivo* Limiting Dilution Analysis for CA2 CCSCs. A. The ADLEFLUOR® (StemCell Technologies) was used to enrich for CCSCs and progenitor cells. Shown is a histogram gated for differential enrichment of ALDEFLUOR^{high} CCSCs and ALDEFLUOR^{low} progenitor cells. **B.** *In vivo* limiting dilution analysis. To confirm self-renewal activity, serial injections of limiting dilutions of ALDEFLUOR^{high} and ALDEFLUOR^{low} subpopulations were injected subcutaneously into the flanks of three NSG mice to generate primary tumor xenografts. Resulting tumors were pooled, re-enriched, and reinjected into the flank of NSG mice to generate secondary tumors. Results are displayed as tumor frequencies in the table and tumor growth is plotted as tumor volume (mm^3) versus time (weeks). Notably, the tumorigenicity curves for these secondary injections revealed significantly fewer successful tumor xenografts with a prolonged tumor latency.

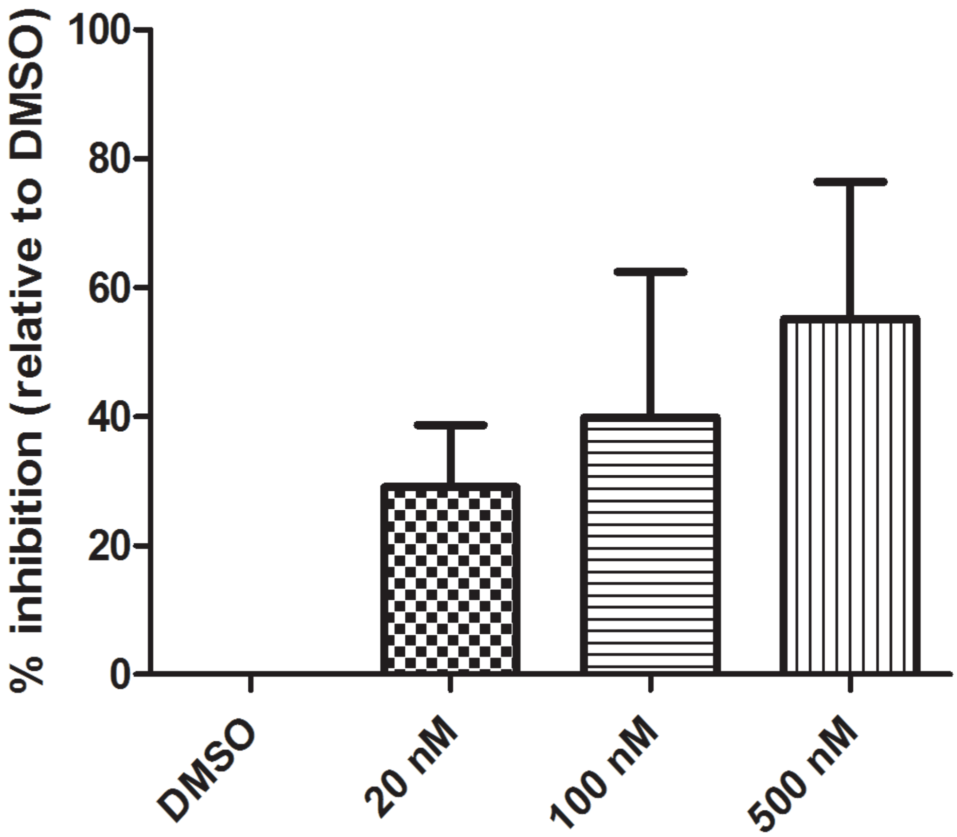

Supplementary Figure S2: Rapamycin, a mTOR inhibitor, exhibits limited inhibition of CCSC proliferation. BrdU proliferation assay in the presence of increasing doses of rapamycin (0-500 nM). Y-axis: relative light units per second, a measure of BrdU incorporation, represented as percent inhibition compared to 0 nM (DMSO) control. X-axis: drug concentrations. (n=2, p=0.1189, one-way ANOVA).

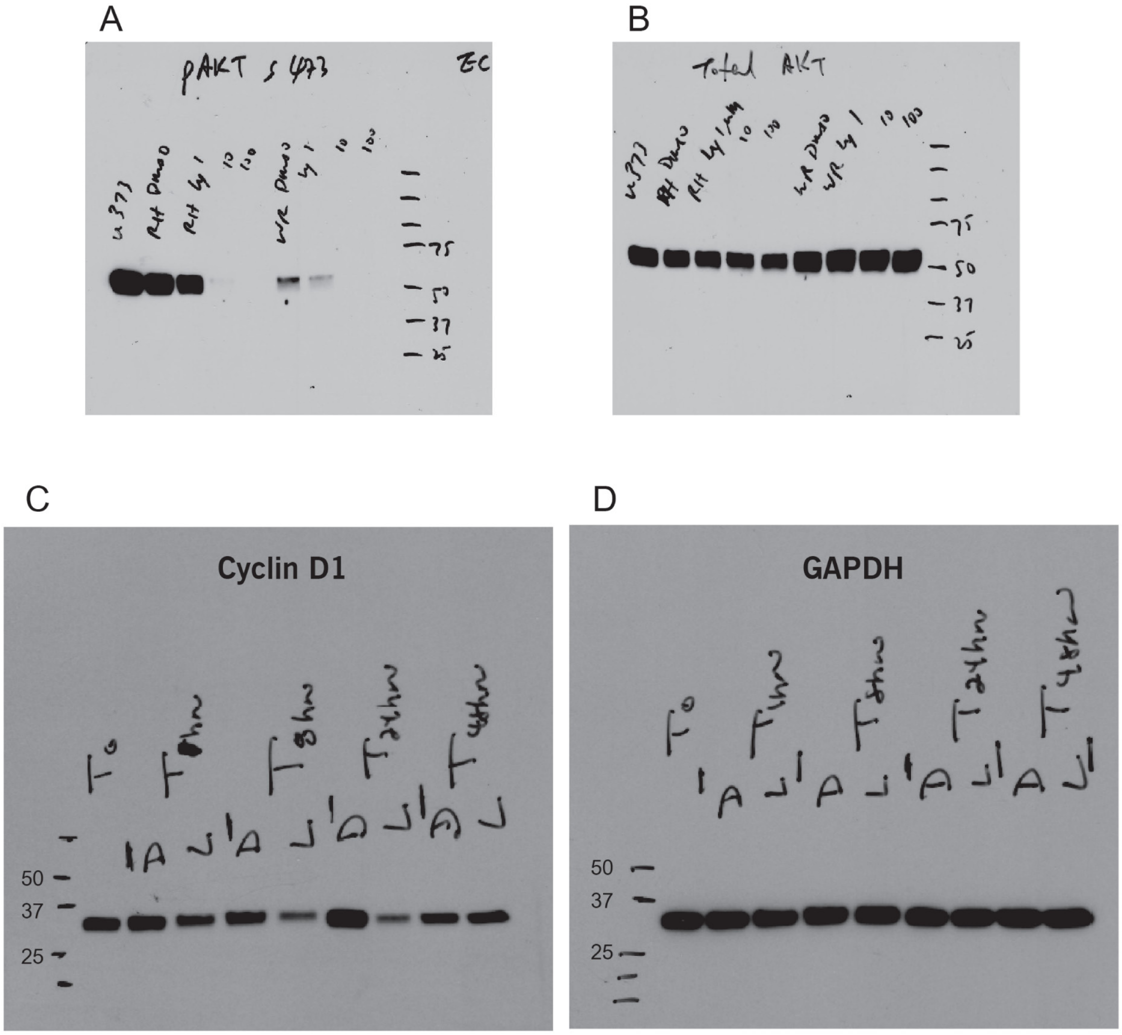

Supplementary Figure S3: A and **B.** Western blot for phosphorylated Akt (Serine 473) and total Akt. Complete images of western blot for treated CA2 CCSCs demonstrates decreased phosphorylation of Akt at serine 473 in the presence of increasing concentrations of the PI3K inhibitor, LY294002. **C** and **D.** Western blot for Cyclin D1 and GAPDH. Complete image of western blots for Ly294002-treated CA2 CCSCs demonstrates decreasing levels of Cyclin D1 at 8 hours and 24 hours.

Supplementary Table S1: Patients and Characteristics

Identification	Gender	Age	Location	Stage
**CA1**	M	74	Rectum	1
**CA2**	F	65	Right	3
**CA7**	F	58	Sigmoid	3
**CA11**	M	50	Right	3
**CA17**	F	51	Right	2
**CA18**	M	79	Rectum	2

Supplementary Table 2: List of 136 Genes Differentially Expressed between ALDEFLUOR^{high} CCSCs and ALDEFLUOR^{low} Progenitors.

See Supplementary File: 1
