# Supplementary material for: Inhibition of PI3K/Akt/mTOR signaling in PI3KR2-overexpressing colon cancer stem cells reduces tumor growth due to apoptosis
Source: Oncotarget. 2016 Jun 8;8(31):50476–88. doi: 10.18632/oncotarget.9919 (PMC5584153; doi:10.18632/oncotarget.9919)
Supplement: Supplementary file 2 [file oncotarget-08-50476-s002.docx]

| order  **Supplemental Table 2. List of 135 Genes Differentially Expressed between ALDEFLUOR^high^ CCSCs and ALDEFLUOR^low^ Progenitors cells.** | Parametric p-value | t-value | ALDEFLUOR^high^ CCSC vs. ALDEFLUOR^low^ Progenitor Fold-Change | Probe set | Gene symbol | Name |
| --- | --- | --- | --- | --- | --- | --- |
| 1 | 2.54E-05 | 5.586 | 7.7 | 57540_at | NA | NA |
| 2 | 3.35E-05 | 5.456 | 7.7 | 205046_at | CENPE | centromere protein E, 312kDa |
| 3 | 4.21E-05 | 5.349 | 8.3 | 225129_at | CPNE2 | copine II |
| 4 | 4.45E-05 | 5.323 | 10.6 | 206102_at | GINS1 | GINS complex subunit 1 (Psf1 homolog) |
| 5 | 6.79E-05 | 5.128 | 8.3 | 201790_s_at | DHCR7 | 7-dehydrocholesterol reductase |
| 6 | 7.02E-05 | 5.112 | 7.7 | 219641_at | DET1 | de-etiolated homolog 1 (Arabidopsis) |
| 7 | 7.21E-05 | 5.1 | 6.3 | 226433_at | RNF157 | ring finger protein 157 |
| 8 | 9.00E-05 | 4.998 | 7.7 | 204346_s_at | RASSF1 | Ras association (RalGDS/AF-6) domain family member 1 |
| 9 | 0.0001022 | 4.94 | 5.9 | 227679_at | HDAC11 | histone deacetylase 11 |
| 10 | 0.0001086 | 4.912 | 6.7 | 205333_s_at | RCE1 | RCE1 homolog, prenyl protein peptidase (S. cerevisiae) |
| 11 | 0.0001286 | 4.835 | 5.9 | 231069_at | NA | NA |
| 12 | 0.0001298 | 4.831 | 6.7 | 209824_s_at | ARNTL | aryl hydrocarbon receptor nuclear translocator-like |
| 13 | 0.0001388 | 4.8 | 9.1 | 212548_s_at | FRYL | FRY-like |
| 14 | 0.0001587 | 4.739 | 7.7 | 211056_s_at | SRD5A1 | steroid-5-alpha-reductase, alpha polypeptide 1 (3-oxo-5 alpha-steroid delta 4-dehydrogenase alpha 1) |
| 15 | 0.0001689 | 4.711 | 6.3 | 214023_x_at | TUBB2B | tubulin, beta 2B |
| 16 | 0.0001854 | 4.669 | 5.9 | 224015_s_at | MRPS25 | mitochondrial ribosomal protein S25 |
| 17 | 0.0001893 | 4.659 | 6.7 | 218782_s_at | ATAD2 | ATPase family, AAA domain containing 2 |
| 18 | 0.0001956 | 4.645 | 5.9 | 215005_at | NECAB2 | N-terminal EF-hand calcium binding protein 2 |
| 19 | 0.0002043 | 4.625 | 5.3 | 227400_at | NFIX | nuclear factor I/X (CCAAT-binding transcription factor) |
| 20 | 0.0002082 | 4.616 | 5.9 | 238013_at | PLEKHA2 | pleckstrin homology domain containing, family A (phosphoinositide binding specific) member 2 |
| 21 | 0.0002229 | 4.585 | 5.3 | 213853_at | DNAJC24 | DnaJ (Hsp40) homolog, subfamily C, member 24 |
| 22 | 0.0002321 | 4.567 | 5 | 222754_at | TRNT1 | tRNA nucleotidyl transferase, CCA-adding, 1 |
| 23 | 0.0002339 | 4.564 | 5.3 | 216239_at | BBS9 | Bardet-Biedl syndrome 9 |
| 24 | 0.0002345 | 4.562 | 6.3 | 232681_at | NA | NA |
| 25 | 0.0002499 | 4.534 | 6.7 | 236126_at | ACVR2B | activin A receptor, type IIB |
| 26 | 0.0002601 | 4.516 | 8.3 | 225012_at | HDLBP | high density lipoprotein binding protein |
| 27 | 0.0002618 | 4.513 | 5.9 | 225044_at | NT5C3L | 5'-nucleotidase, cytosolic III-like |
| 28 | 0.0002975 | 4.455 | 6.7 | 224441_s_at | USP45 | ubiquitin specific peptidase 45 |
| 29 | 0.0003163 | 4.428 | 6.3 | 209903_s_at | ATR | ataxia telangiectasia and Rad3 related |
| 30 | 0.0003232 | 4.418 | 4.8 | 207165_at | HMMR | hyaluronan-mediated motility receptor (RHAMM) |
| 31 | 0.000331 | 4.407 | 5.9 | 228151_at | NA | NA |
| 32 | 0.0003316 | 4.407 | 4.8 | 211330_s_at | HFE | hemochromatosis |
| 33 | 0.0003525 | 4.379 | 6.3 | 235039_x_at | LIN9 | lin-9 homolog (C. elegans) |
| 34 | 0.0003955 | 4.327 | 5.9 | 202049_s_at | ZMYM4 | zinc finger, MYM-type 4 |
| 35 | 0.0003977 | 4.325 | 4.8 | 235117_at | CHAC2 | ChaC, cation transport regulator homolog 2 (E. coli) |
| 36 | 0.0004388 | 4.281 | 7.1 | 225236_at | RBM18 | RNA binding motif protein 18 |
| 37 | 0.0004498 | 4.27 | 8.3 | 204866_at | PHF16 | PHD finger protein 16 |
| 38 | 0.0004666 | 4.253 | 5.9 | 210758_at | PSIP1 | PC4 and SFRS1 interacting protein 1 |
| 39 | 0.0004736 | 4.247 | 5 | 229615_at | NA | NA |
| 40 | 0.0004769 | 4.244 | 4.8 | 218925_s_at | C11orf1 | chromosome 11 open reading frame 1 |
| 41 | 0.0004904 | 4.231 | 4.5 | 220201_at | RC3H2 | ring finger and CCCH-type zinc finger domains 2 |
| 42 | 0.0005203 | 4.205 | 4.5 | 208353_x_at | ANK1 | ankyrin 1, erythrocytic |
| 43 | 0.0005288 | 4.197 | 5 | 202932_at | YES1 | v-yes-1 Yamaguchi sarcoma viral oncogene homolog 1 |
| 44 | 0.0005308 | 4.196 | 4.8 | 213138_at | ARID5A | AT rich interactive domain 5A (MRF1-like) |
| 45 | 0.0005319 | 4.195 | 5 | 217905_at | C10orf119 | chromosome 10 open reading frame 119 |
| 46 | 0.0005417 | 4.187 | 5.6 | 223493_at | FBXO4 | F-box protein 4 |
| 47 | 0.0005476 | 4.182 | 10 | 238183_at | NA | NA |
| 48 | 0.0005488 | 4.181 | 4.5 | 223093_at | ANKH | ankylosis, progressive homolog (mouse) |
| 49 | 0.0005587 | 4.173 | 4.5 | 203022_at | RNASEH2A | ribonuclease H2, subunit A |
| 50 | 0.0005699 | 4.164 | 8.3 | 235767_x_at | NA | NA |
| 51 | 0.0005794 | 4.156 | 5.6 | 227986_at | ZNF343 | zinc finger protein 343 |
| 52 | 0.0006348 | 4.116 | 5.9 | 223268_at | C11orf54 | chromosome 11 open reading frame 54 |
| 53 | 0.0006516 | 4.104 | 5 | 204354_at | POT1 | POT1 protection of telomeres 1 homolog (S. pombe) |
| 54 | 0.0006537 | 4.103 | 6.3 | 221654_s_at | USP3 | ubiquitin specific peptidase 3 |
| 55 | 0.0006734 | 4.089 | 5.6 | 219021_at | RNF121 | ring finger protein 121 |
| 56 | 0.0006938 | 4.076 | 4.3 | 203755_at | BUB1B | budding uninhibited by benzimidazoles 1 homolog beta (yeast) |
| 57 | 0.0007147 | 4.063 | 5 | 207513_s_at | ZNF189 | zinc finger protein 189 |
| 58 | 0.0007214 | 4.058 | 5.6 | 225583_at | UXS1 | UDP-glucuronate decarboxylase 1 |
| 59 | 0.0007229 | 4.058 | 4.5 | 202331_at | BCKDHA | branched chain keto acid dehydrogenase E1, alpha polypeptide |
| 60 | 0.0007256 | 4.056 | 5.9 | 222848_at | CENPK | centromere protein K |
| 61 | 0.0007262 | 4.056 | 5.9 | 225252_at | SRXN1 | sulfiredoxin 1 homolog (S. cerevisiae) |
| 62 | 0.0007326 | 4.052 | 5.9 | 225355_at | NEURL1B | neuralized homolog 1B (Drosophila) |
| 63 | 0.0007461 | 4.043 | 4 | 217244_at | NA | NA |
| 64 | 0.0007493 | 4.042 | 4.3 | 225108_at | AGPS | alkylglycerone phosphate synthase |
| 65 | 0.0008028 | 4.011 | 4.3 | 224308_s_at | INTS2 | integrator complex subunit 2 |
| 66 | 0.0008266 | 3.998 | 6.3 | 204566_at | PPM1D | protein phosphatase 1D magnesium-dependent, delta isoform |
| 67 | 0.0008616 | 3.979 | 4 | 226272_at | RCAN3 | RCAN family member 3 |
| 68 | 0.0008932 | 3.963 | 4.5 | 217668_at | C22orf36 | chromosome 22 open reading frame 36 |
| 69 | 0.0008946 | 3.962 | 4.8 | 202457_s_at | PPP3CA | protein phosphatase 3 (formerly 2B), catalytic subunit, alpha isoform |
| 70 | 0.0008965 | 3.961 | 4 | 226731_at | PELO | pelota homolog (Drosophila) |
| 71 | 0.000905 | 3.957 | 5.3 | 229392_s_at | PIK3R2 | phosphoinositide-3-kinase, regulatory subunit 2 (beta) |
| 72 | 0.0009147 | 3.952 | 5.9 | 224883_at | PLDN | pallidin homolog (mouse) |
| 73 | 0.0009155 | 3.952 | 4.8 | 204675_at | SRD5A1 | steroid-5-alpha-reductase, alpha polypeptide 1 (3-oxo-5 alpha-steroid delta 4-dehydrogenase alpha 1) |
| 74 | 0.0009238 | 3.948 | 5.6 | 226319_s_at | THOC4 | THO complex 4 |
| 75 | 0.0009319 | 3.944 | 5.6 | 38157_at | DOM3Z | dom-3 homolog Z (C. elegans) |
| 76 | 0.0009389 | 3.941 | 5.9 | 223171_at | DYM | dymeclin |
| 77 | 0.0009654 | 3.928 | 5.9 | 215726_s_at | CYB5A | cytochrome b5 type A (microsomal) |
| 78 | 0.0009899 | -3.917 | -4.9 | 219279_at | DOCK10 | dedicator of cytokinesis 10 |
| 79 | 0.0009625 | -3.93 | -4 | 241464_s_at | NA | NA |
| 80 | 0.0009524 | -3.934 | -5.3 | 1560322_at | RBMS3 | RNA binding motif, single stranded interacting protein |
| 81 | 0.0009107 | -3.954 | -4.1 | 1565898_at | METT5D1 | methyltransferase 5 domain containing 1 |
| 82 | 0.0009048 | -3.957 | -4.4 | 211187_at | NA | NA |
| 83 | 0.0008953 | -3.962 | -5.1 | 242194_at | CUL4A | cullin 4A |
| 84 | 0.000891 | -3.964 | -4.4 | 1569941_at | NA | NA |
| 85 | 0.0008745 | -3.973 | -4.3 | 235954_at | NA | NA |
| 86 | 0.0008495 | -3.986 | -5 | 1569203_at | CXCL2 | chemokine (C-X-C motif) ligand 2 |
| 87 | 0.0007934 | -4.016 | -4.7 | 236567_at | NA | NA |
| 88 | 0.0007928 | -4.016 | -5.4 | 214825_at | FAM155A | family with sequence similarity 155, member A |
| 89 | 0.0007588 | -4.036 | -4.9 | 214667_s_at | TP53I11 | tumor protein p53 inducible protein 11 |
| 90 | 0.0007582 | -4.036 | -6.2 | 229279_at | NA | NA |
| 91 | 0.000722 | -4.058 | -4.5 | 224211_at | FOXP3 | forkhead box P3 |
| 92 | 0.0006993 | -4.072 | -4.4 | 230662_at | RNF187 | ring finger protein 187 |
| 93 | 0.0006716 | -4.09 | -5.1 | 207774_at | ATG10 | ATG10 autophagy related 10 homolog (S. cerevisiae) |
| 94 | 0.0006598 | -4.098 | -4.3 | 236301_at | NA | NA |
| 95 | 0.0006595 | -4.099 | -4.2 | 1566783_at | NA | NA |
| 96 | 0.0006445 | -4.109 | -4.2 | 204374_s_at | GALK1 | galactokinase 1 |
| 97 | 0.0006339 | -4.116 | -5.1 | 232980_at | NA | NA |
| 98 | 0.0006332 | -4.117 | -6.1 | 244364_at | MYO3A | myosin IIIA |
| 99 | 0.00062 | -4.126 | -4.3 | 1559410_at | NA | NA |
| 100 | 0.0005942 | -4.145 | -5.2 | 1561099_at | FLJ32756 | hypothetical LOC642757 |
| 101 | 0.0005732 | -4.161 | -5.4 | 224062_x_at | KLK4 | kallikrein-related peptidase 4 |
| 102 | 0.0005302 | -4.196 | -7.1 | 242377_x_at | THUMPD3 | THUMP domain containing 3 |
| 103 | 0.0004893 | -4.232 | -5.2 | 230936_at | DNAJB13 | DnaJ (Hsp40) related, subfamily B, member 13 |
| 104 | 0.0004815 | -4.239 | -4.5 | 230489_at | CD5 | CD5 molecule |
| 105 | 0.0004456 | -4.274 | -6.2 | 238611_at | NA | NA |
| 106 | 0.0004453 | -4.274 | -7.1 | 229772_at | DEFB123 | defensin, beta 123 |
| 107 | 0.0004266 | -4.293 | -6.5 | 215560_x_at | MTRF1L | mitochondrial translational release factor 1-like |
| 108 | 0.0004259 | -4.294 | -4.2 | 1557404_at | NA | NA |
| 109 | 0.0004241 | -4.296 | -5.8 | 1556445_at | NA | NA |
| 110 | 0.0004224 | -4.298 | -4.6 | 1560358_at | PHKA2 | phosphorylase kinase, alpha 2 (liver) |
| 111 | 0.0004015 | -4.321 | -5.6 | 211305_x_at | FCAR | Fc fragment of IgA, receptor for |
| 112 | 0.0003996 | -4.323 | -7 | 1563853_at | LOC283045 | hypothetical protein LOC283045 |
| 113 | 0.0003942 | -4.329 | -4.7 | 216810_at | KRTAP4-7 | keratin associated protein 4-7 |
| 114 | 0.0003802 | -4.345 | -4.6 | 240971_x_at | NA | NA |
| 115 | 0.0003714 | -4.356 | -4.4 | 1562742_at | NA | NA |
| 116 | 0.0003412 | -4.394 | -5.7 | 213515_x_at | NA | NA |
| 117 | 0.0003328 | -4.405 | -4.7 | 218736_s_at | PALMD | palmdelphin |
| 118 | 0.0003205 | -4.422 | -5.3 | 239195_at | NA | NA |
| 119 | 0.0002692 | -4.5 | -5.9 | 214414_x_at | NA | NA |
| 120 | 0.0002395 | -4.553 | -8 | 236437_at | NA | NA |
| 121 | 0.0002279 | -4.575 | -6.8 | 209116_x_at | HBB | hemoglobin, beta |
| 122 | 0.0002057 | -4.622 | -5.8 | 241965_at | NA | NA |
| 123 | 0.0001931 | -4.65 | -6.1 | 215199_at | CALD1 | caldesmon 1 |
| 124 | 0.0001908 | -4.656 | -5 | 241181_x_at | NA | NA |
| 125 | 0.0001731 | -4.7 | -5 | 241530_at | NA | NA |
| 126 | 0.0001612 | -4.732 | -6.1 | 1561323_at | LOC339975 | hypothetical protein LOC339975 |
| 127 | 0.0001573 | -4.743 | -5.2 | 1566908_at | LOC441476 | hypothetical protein LOC441476 |
| 128 | 0.0001261 | -4.844 | -7 | 217557_s_at | CPM | carboxypeptidase M |
| 129 | 0.000106 | -4.923 | -8.2 | 217683_at | HBE1 | hemoglobin, epsilon 1 |
| 130 | 7.43E-05 | -5.086 | -7.2 | 232557_at | LOC390595 | similar to ubiquitin-associated protein 1 (predicted) |
| 132 | 7.20E-05 | 5.127 | -6.3 | 1563210_at | NA | NA |
| 133 | 6.80E-05 | -5.127 | -8.1 | 1556287_a_at | NA | NA |
| 134 | 6.74E-05 | -5.131 | -9.5 | 217572_at | NA | NA |
| 135 | 3.10E-05 | -5.493 | -11.8 | 211745_x_at | NA | NA |
| 136 | 4.50E-06 | -6.429 | -9.5 | 220954_s_at | PILRB | paired immunoglobin-like type 2 receptor beta |

**Supplemental Table 2**. List of 136 Genes Differentially Expressed between ALDEFLUOR^high^ CCSCs and ALDEFLUOR^low^ Progenitors. The top 136 differential expressed genes between ALDEFLUOR^high^ CCSCs and ALDEFLUOR^low^ progenitors as defined by a paired t-test (p<0.001) are listed. For each gene, parametric p value, t value, fold-change and Affymetrix probe set are indicated. NA denotes not mapping to a known gene symbol.
